# Electrical Excitation of Long-Range Surface Plasmons in PC/OLED Structure with Two Metal Nanolayers

**DOI:** 10.1007/s40820-020-0369-7

**Published:** 2020-01-22

**Authors:** Valery Konopsky, Valery Prokhorov, Dmitry Lypenko, Artem Dmitriev, Elena Alieva, Giovanni Dietler, Sergey Sekatskii

**Affiliations:** 1 Institute of Spectroscopy, Russian Academy of Sciences, Fizicheskaya, 5, Troitsk, Moscow, Russia 108840; 2 Frumkin Institute of Physical Chemistry and Electrochemistry Russian Academy of Sciences, Leninsky pr., 31/4, Moscow, Russia 119071; 3 Laboratoire de Physique de La Matière Vivante, IPHYS, Ecole Polytechnique Fédérale de Lausanne, 1015 Lausanne, Switzerland

**Keywords:** Surface plasmons, Photonic crystal waveguides, Light-emitting polymers

## Abstract

A current-driven source of long-range surface plasmons (LRSPs) on a duplex metal nanolayer is reported. Electrical excitation of LRSPs was experimentally observed in a planar structure, where an organic light-emitting film was sandwiched between two metal nanolayers that served as electrodes. To achieve the LRSP propagation in these metal nanolayers at the interface with air, the light-emitting structure was bordered by a one-dimensional photonic crystal (PC) on the other side. The dispersion of the light emitted by such a hybrid PC/organic-light-emitting-diode structure (PC/OLED) comprising two thin metal electrodes was obtained, with a clearly identified LRSP resonance peak.

## Highlights

- Long-range surface plasmons were first excited in a hybrid photonic-crystal/organic-light-emitting-diode microstructure containing two metal nanolayers.
- These surface plasmons were excited without any external laser light, but by injecting current through the two metal nanolayers, which serve as thin metal electrodes for organic light-emitting microfilm between the layers.

## Graphical abstract

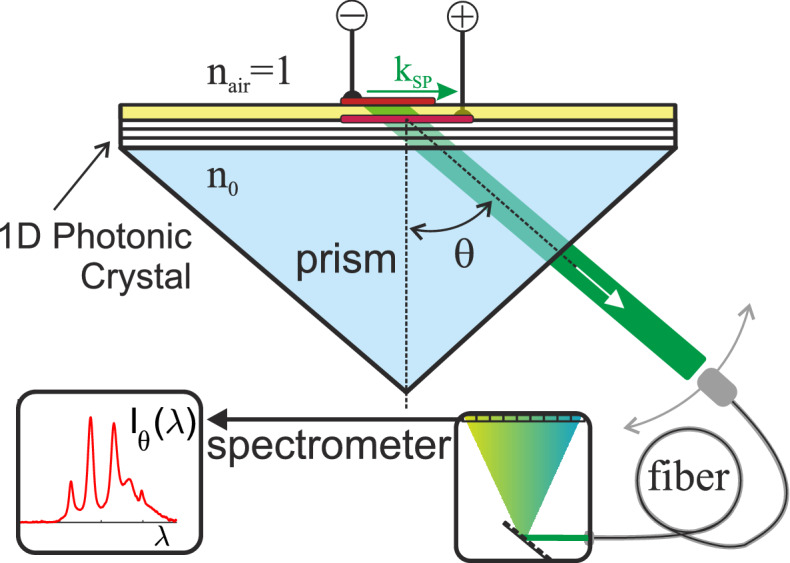

## Introduction

The broad interest in the problems associated with surface plasmon (SP) amplification and lasing, witnessed for at least 20 years, has been driven by the practical necessities of the miniaturization of electronic and optical devices: lowering power consumption, increasing the operating frequency range, requiring increasing degrees of integration. There is a general belief that optics-based approaches, due to their inherent advantages over electronic ones, will progressively replace the latter. The twenty-first century has often been referred to as the “century of photons” in the same sense that the previous century was that of electrons. Profound scientific studies and technological research in the field of surface plasmon lasing, starting with the SPASER proposal in 2003 [1] and the subsequent experimental realization of such, or similar, devices [2–10], are essential steps in this direction.

However, without the denial of substantial and real progress, two requirements (which, as of today, have been seldom met) should be emphasized. First, any surface source of light that can work in the field of future photonics or its interface with electronics must not depend on an external bulk laser (for corresponding operational purposes) providing optical pumping. Second, the generation of surface plasmons propagating exactly along the corresponding interface(s), rather than, for example, lasing in the direction perpendicular to the surface with the nanoplasmonic particles/structures, is needed.

**Fig. 1:**
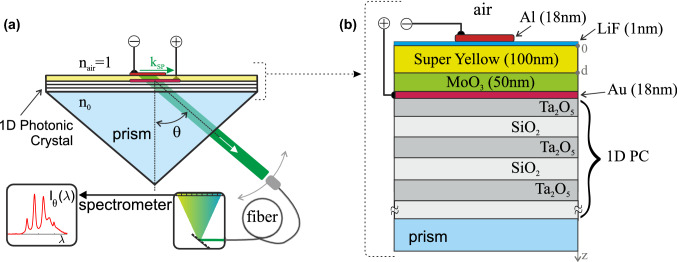
**a** Layout of the experiment and **b** the PC/OLED structure with two metal nanolayers

In this work our first results with regard to the fabrication of such devices are reported. In the system under study, a current injection-driven organic light-emitting diode (OLED) is sandwiched between two thin metal layers (Au and Al electrodes) and deposited on a 1D photonic crystal (PC). Thus, the complete multilayer structure, shown in detail in Fig. 1, comprises the following layers: [PC/M_1_/OLED/M_2_/air]. The integral thickness of the OLED is selected such that *both* metal electrodes, if thin enough, can support the long-range propagation of surface plasmons.

The ability of a *single* metal nanolayer to support LRSP propagation in a symmetric dielectric/thin-metal-layer/dielectric [D/M/D] structure has been well known since the early 1980s [11, 12] and is widely used in plasmonics [13]. A multilayer structure in the form [PC/M/air] has been proposed to simplify the implementation of LRSP in practical applications, where the external dielectric is air [14]. This approach was tested with diverse systems, including thin palladium layers (for ultrasensitive hydrogen detection [15–17]), thin gold layers in blue spectral range (for nitrogen dioxide detection [18]), and thin ferromagnetic cobalt layers (for magnetoplasmonics [19]), among others (see also [20–23] for examples of other applications).

However, in the works mentioned above, structures comprising only one thin metal layer were exploited. Recently, it has been recognized that *two* thin metal layers, with appropriate dielectric spacing between them, also can quite effectively support long-range surface plasmon (LRSP) propagation [24]. This design has implemented in the present study and the current injection in the OLED is caused by the (DC) voltage applied between these two (possibly thin) metal electrodes.

## Plasmons in Duplex Metal Layer

### Experimental Setup

The experimental setup is presented in Fig. 1a. The structure in the study [PC/Au/OLED/Al/air] was placed onto the quartz right-angle prism’s hypotenuse. A thin layer of immersion oil was used to attain optical continuity and refractive index (RI) matching between the substrate of the PC chip and the prism. The light emitted from the multilayer structure was collected via a multimode optical fiber with the input face rigidly attached to the rotating arm of the homemade screw-gear setup, allowing angular scanning with an accuracy of 0.25°. At each fixed registration angle, the spectrum of the collected light was recorded using the AvaSpec 2048 fiber-optic spectrophotometer (Netherlands), with a spectral resolution 0.04 nm.

### 1D Photonic Crystal

The 1D PC part of the multilayer structure was deposited by magnetron sputtering and has the form [PC] = [substrate/(*H*·*L*)^*N*^/*H*′], where *L* is a SiO_2_ layer with thickness *d*_1_ = 118.6 nm, *H* is a Ta_2_O_5_ layer with *d*_2_ = 86.8 nm, and *H*′ is a Ta_2_O_5_ layer with *d*_3_ = 75.8 nm. The prism and the substrate were made from fused silica. The RI of the Ta_2_O_5_ layers is *n*_2_ = *n*_3_ = 2.11, whereas the RIs of the substrate and prism and SiO_2_ layers are *n*_0_ = *n*_1_ = 1.46 (at wavelength *λ* = 575 nm). When *N* = 13 (in the present case), this SiO_2_/Ta_2_O_5_ 27-layer structure (started and finished with Ta_2_O_5_ layers) permits guided waves, propagating along the outer layers, to be decoupled through a prism at a resonance angle *θ*, as shown in Fig. 1a. Thus, this scheme can be called an inverted Kretschmann geometry. If the number *N* increases, this decoupling pathway vanishes, and only emissions through the edges of this multilayer structure are possible.

### OLED Composition

The OLED portion of the multilayer structure has the form: [OLED] = [MoO_3_/SY/LiF], with the thicknesses of the layers shown in Fig. 1b. The RIs of the OLED layers, at *λ* = 575 nm, are 2.0 for the transport layer (MoO_3_), 1.86 for the light-emitting layer (SY), and 1.39 for the barrier layer (LiF). The RIs of Au and Al at this wavelength are *n*_Au_ = 0.3 + 2.8*i* and *n*_Al_ = 1.1 + 6.9*i*, respectively [25–27]. The maximum brightness of a test OLED structure with the parameters mentioned above, but without the 1D PC section [glass/Au/OLED/Al/air], measured in the direction perpendicular to the layers, was 13,500 cd m^−2^ at 12 V.

**Fig. 2:**
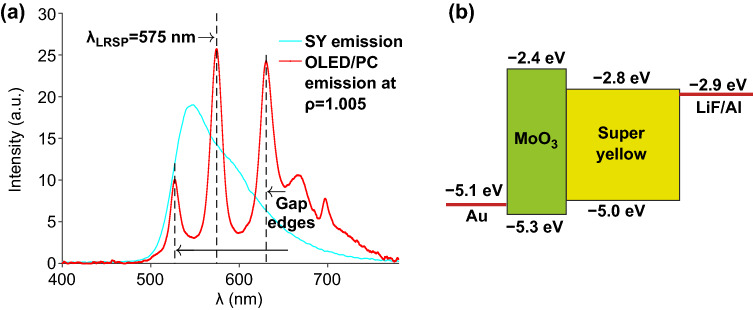
**a** Electroluminescence spectrum from the 1D PC with the Super Yellow light-emitting layer, which is sandwiched between two metal nanolayers. The standard Super Yellow emission spectrum is presented as a cyan curve for comparison. **b** The energy level diagram of the OLED layers. (Color figure online)

Super Yellow (SY, PDY-132, Merck; *M*_*w*_ > 1,300,000, *M*_*n*_ > 200,000) is an efficient electroluminescent material having a broad luminescence band centered around 570 nm; its emission spectrum is presented in Fig. 2a as a cyan line. This material is widely used as a polymer light-emitting layer for OLED manufacturing due to its high stability and excellent brightness characteristics [28, 29]. The technical reason for choosing MoO_3_ (Lumtec, Taiwan) as the material for the hole injection-transport layer (HTL) is that it does not dissolve in chlorobenzene that is used for the structure preparation, namely during the spin-coating deposition of the SY layer slightly above the transport layer. Besides this technical reason, MoO_3_ has quite suitable alignment of energy levels for the band structure of the active layer and Au anode [30]. The energy level diagram of the OLED layers is presented in Fig. 2b. The position of the HTL energy levels is critical for OLED functioning, making it possible to reduce the drive voltage by enhancing the charge injection at the interface, thereby improving the power efficiency of the device.

### OLED Fabrication on the 1D PC

The OLED manufacturing process was as follows. First, the surface of the 1D PC structure was irradiated by an ultraviolet lamp for 15–20 min to clean and activate the external PC surface. Next, the auxiliary 90-nm-thick layer of Al was deposited at a rate of 0.02–0.04 nm s^−1^ through the specially designed mask onto a part of the PC surface by thermal evaporation in vacuum at a pressure of 6 × 10^–6^ Tor. This thick metal layer surrounding the OLED area was used for the subsequent attachment of external electrical contacts (electrodes). Then, the 18-nm-thick gold layer (OLED anode) was deposited through another mask by thermal evaporation using the same vacuum and rate conditions. This layer, at certain places predefined by the mask design, physically and electrically contacts the previously deposited thick Al layer and, therefore, via this layer, also with the external electrodes. Four different independent OLED structures with sizes of approximately 4 × 4 mm^2^ were prepared on each 1D PC chip with a diameter of 25.4 mm.

Furthermore, a 50-nm-thick MoO_3_ transport layer was deposited with a rate of 0.03 nm s^−1^ in the same vacuum conditions. The next step was the spin-coating deposition of the SY layer over the MoO_3_ layer. Then, 80–90 µL of a well-agitated solution of SY in chlorobenzene, with a concentration of 5 mg mL^−1^, was used for the spin coating at a rotation speed of 1000 rpm and approximately 1-min exposition time. Then, the sample was allowed to dry for 12 h at room temperature and for 4 h at 800 °C to remove the residual chlorobenzene traces. The OLED samples were prepared, and their spectral and photoelectric characteristics were measured at room temperature in a glove box (MBraun, Germany) under an argon atmosphere with a controlled content of oxygen and water (below 1 ppm). Finally, 1-nm-thick LiF and 18-nm-thick Al (cathode) layers were thermally deposited, again in the same vacuum and rate conditions as described above, with the use of the third mask. Voltage–current and voltage–brightness characteristics were measured with a Keithley 2601 SourceMeter, Keithley (USA) 485 pico-ammeter and TKA-04/3 luxmeter-brightness meter (Russia). The thicknesses of the films were determined using an MII-4 interferometer (LOMO, Saint Petersburg, Russia).

## Results and Discussion

### LRSP Resonance Observation

The red curve in Fig. 2a shows a representative example of the recorded spectra at registration angle $\rho = n_0\sin(\theta )=1.005$. The LRSP resonance is seen at ${\lambda }_{\mathrm{LRSP}} =575$ nm, while two local maxima at 527 and 630 nm correspond to the bandgap edges of the structure under study. Such measurements were repeated point-by-point across the angular parameters *ϱ* = 0.89…1.12. Due to the finite sizes of the light source and the optical fiber input aperture, the recorded spectra roughly correspond to a 2° convolution around the selected angle.

**Fig. 3:**
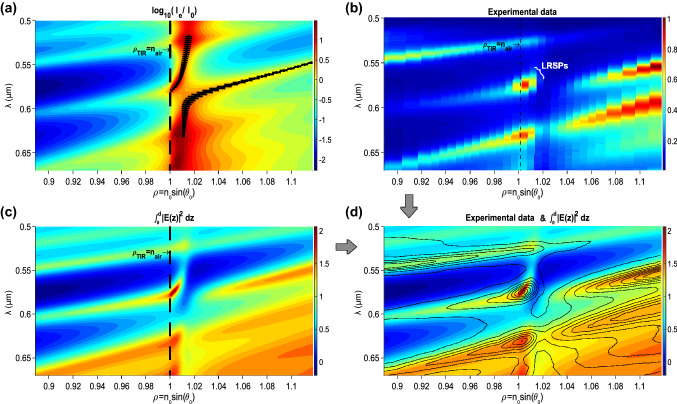
Dispersion plots of the light-emitting structure under study: **a** calculated intensity enhancement at the external surface (in a logarithmic color scale), and the dispersion curves of surface modes for a semi-infinite 1D PC (black lines, the error bar represents the imaginary part of *ϱ*), **b** the stack of 29 experimental spectra of electroluminescence taken at different angular parameters *ϱ*, **c** calculated integral of the optical electric field in the Super Yellow layer, and **d** superimposed experimental spectra (as smoothed contour lines) and the integral of the optical electric field in the SY layer. (Color figure online)

The stacked set of such angular measurements taken at different *ϱ* values provides a two-dimensional picture *I*(*ϱ*,*λ*), shown in Fig. 3b, which reveals a local maximum at the point (1.003, 575 nm), corresponding to the LRSP excitation. It was previously demonstrated [14, 31] that the excitation of optical surface waves with an effective RI near

1 $${\rho }_{1/2}={n}_{e}+\frac{{n}_{\mathrm{e}}^{3}}{2}{\left[\pi \frac{{{d}}_{\mathrm{m}}}{\lambda }\right]}^{2}$$

is an essential prerequisite for the LRSP propagation along a thin metal film. For the classical LRSP structure [D/M/D], this condition is satisfied automatically [31, 32]. In theoretical work [24], it was found that the same prerequisite holds for structures containing two metal nanolayers. In this case, the tangential component of the electric field is zero at the centers of *both* metallic nanofilms, and the losses of optical waves are small. In the experiments, both metal nanolayers had a thickness of *d*_m_ = 18 nm, and from Eq. (1) one obtains *ϱ*_1/2_ = 1.003 (for *λ* = 575 nm and *n*_*e*_ $\simeq$ 1). Thus, the experimental value given above agrees well with this value.

### Dispersion Plots

Figure 3 demonstrates two-dimensional dispersion plots of the light-emitting structure under study in coordinates (*ϱ*, *λ*). In Fig. 3a, the magnitude of the optical-field enhancement near the external interface with the air is depicted in color tones with the logarithmic color scale (shown to the right). This field enhancement was calculated for a real structure containing the 27 layers of SiO_2_/Ta_2_O_5_ in the 1D PC. The two black curves are the dispersion curves that were determined for a structure with a semi-infinite 1D PC (see work [24] for more details).

Figure 3a shows that two corresponding modes display anticrossing, and one mode is shifted to the light line—shown as the dashed line of total internal reflection ${\rho }_{\mathrm{TIR}}$. The error bar represents the imaginary part of the effective RI *ϱ*, which decreases when the LRSP curve approaches the light line at the point (1.003, 575 nm).

**Fig. 4:**
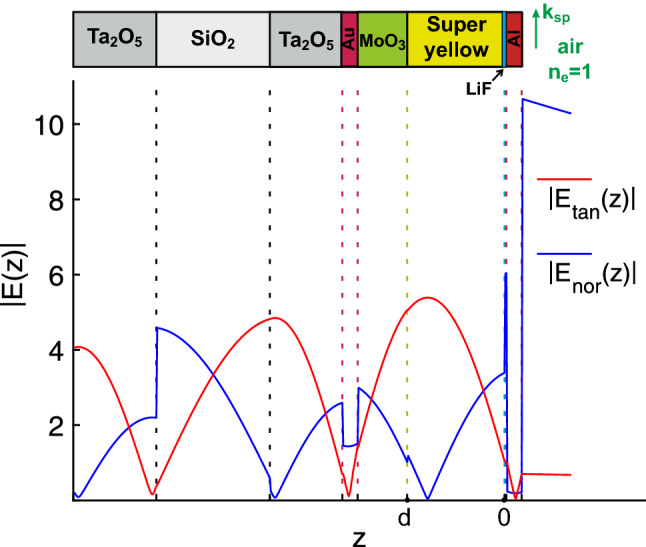
Spatial distribution of the optical-field components in the outer layers of the structure, at *λ* = 575 nm and *ϱ* = 1.003

The reciprocity theorem [33, 34] was used to simulate the experimentally recorded dispersion of electroluminescence, which is shown in Fig. 3b (and which was certainly recorded in the far-field zone). In the present case, it means that the optical electric field, created in the far-field zone by dipoles located in the SY layer—with coordinates from 0 to d, as in Figs. 1b and 4—is the same as the electric field of dipoles from the far-field zone (i.e., from plane optical waves) created in the layer [0 *d*]. The integral of intensity distribution in the SY layer (when the structure is excited by plane waves at different angles of incidence *ϱ* and wavelengths *λ*) is shown in Fig. 3c in color tones. Figure 3d depicts the superposition of the calculated integral of the optical electric field in the Super Yellow (SY) layer, from Fig. 3c, and the experimental spectra, from Fig. 3b (depicted as a smoothed contour plot), with a good agreement between these two figures.

### Field Profiles

The electric-field profiles in the outer layers of the structure under study are shown in Fig. 4. These profiles were calculated for LRSP excitation at *λ* = 575 nm and *ϱ* = 1.003. The tangential component of the optical field is indeed equal to zero at the centers of both metal nanolayers (as it should be at *ϱ*_1/2_), and the total optical field in the metal reaches a minimum with these parameters. This approach ensures minimal energy loss in the metal and maximum propagation length. Notably, in contrast to hybrid dielectric-loaded plasmonic waveguides [35, 36], which are created to achieve the best trade-off between the mode confinement and propagation loss, our multilayer system is designed to achieve minimal propagation loss (even at the cost of the mode confinement). More information on designing LRSP-supporting structures with two metal layers can be found elsewhere [24].

## Conclusions

A current-driven source of LRSPs in a multilayer structure containing an OLED, bounded by two metal nanolayers, on top of a one-dimensional photonic crystal was developed. Electroluminescence spectra were recorded at various decoupling angles, which provided the dispersion profile of the system under study. The LRSP resonance manifested itself at *λ* = 575 nm, near the light line, as expected from the structure design.

The obvious next step is an attempt to achieve LRSP amplification and lasing in the structure with two metal nanolayers. Lasing in the classical symmetric LRSP-supporting structure with one metal nanolayer has been reported for InGaAs quantum-well gain media [37]. However, this was regarding a device with optical pumping, as all SPASERs are today, to the best of the authors’ knowledge (for review of amplification and lasing in LRSP-supporting systems, see [38]). It is hoped that the presented approach will lead to electrical pumping in future SPASERs. Lasing in organic media is itself a challenge; nevertheless, there has been a recent report on current-injection lasing from an organic semiconductor [39], indicating that even an organic current-injection SPASER can be potentially possible.
